# Digital health technologies and measurable management outcomes in primary care: A systematic review, descriptive quantitative synthesis, and temporal sensitivity analysis

**DOI:** 10.1371/journal.pdig.0001778

**Published:** 2026-11-04

**Authors:** William Villegas-Ch, Alejandro Cabrera-Andrade, Diego Buenaño-Fernández, Ana Karina Zambrano

**Affiliations:** 1 Escuela de Ingeniería en Ciberseguridad, FICA, Universidad de Las Américas, Quito, Ecuador; 2 Carrera de Enfermería, Facultad de Ciencias de la Salud, Universidad de Las Américas, Quito, Ecuador; 3 Grupo de Bio-Quimioinformática, Universidad de Las Américas, Quito, Ecuador; 4 Universidad UTE, Facultad de Ciencias de la Salud Eugenio Espejo, Centro de Investigación Genética y Genómica, Quito, Ecuador; Massachusetts Institute of Technology, UNITED STATES OF AMERICA

## Abstract

The rapid digital transformation of primary health care has integrated electronic health records (EHRs), telemedicine, mobile health (mHealth), artificial intelligence (AI), machine learning (ML), and natural language processing (NLP) into routine service management. However, evidence on their relationship with operational efficiency and management performance remains fragmented. This systematic review synthesized evidence on digital health technologies and measurable management outcomes in primary care, including waiting times, administrative efficiency, resource utilization, operating costs, and patient or professional satisfaction. Following PRISMA 2020 principles and the PICO framework, searches were conducted in PubMed/MEDLINE, Scopus, Web of Science, CINAHL, and IEEE Xplore for records published from January 1, 2018, to October 7, 2025. A structured eligibility audit reduced the candidate analytical corpus from 256 records to 172 studies included in the final descriptive quantitative synthesis. Telemedicine/mHealth was the largest category (n = 98; 57.0%), followed by EHR/interoperability (n = 35; 20.3%) and AI/ML or predictive analytics (n = 29; 16.9%). The most frequent indicators were patient or professional satisfaction (n = 24) and operating costs (n = 20). Because of methodological heterogeneity, formal meta-analysis was not performed. Temporal sensitivity analysis showed continuity in EHR/interoperability and telemedicine/mHealth, with a recent increase in AI/ML representation. A stratified preliminary JBI check in 40 studies found mostly low or moderate risk classifications, but it was used only to contextualize reporting quality and does not represent a full risk-of-bias assessment of all 172 studies.

## Author summary

Digital technologies are increasingly used in primary care, but their management impact is difficult to compare across studies because they use different indicators and reporting formats. We reviewed evidence from five databases and analyzed a final cleaned corpus of 172 studies. Telemedicine/mHealth was the most common category, followed by EHR/interoperability and AI/ML/predictive analytics. Patient or professional satisfaction and operating costs were the most frequently reported indicators, whereas waiting times, administrative efficiency, and resource utilization were measured less consistently. Because the evidence was heterogeneous, we used a descriptive quantitative synthesis rather than a meta-analysis, complemented by a temporal sensitivity analysis and a Joanna Briggs Institute (JBI) subsample risk-of-bias check. Stronger evidence will require standardized indicators, transparent reporting, bias-aware evaluation, and prospective real-world studies.

## 1. Introduction

The digital transformation of primary care has changed the way health services are organized, coordinated, and evaluated. Electronic health records (EHRs), interoperable information systems, telemedicine, mobile health (mHealth), artificial intelligence (AI), machine learning (ML), and natural language processing (NLP) are increasingly embedded in routine service delivery. These technologies are no longer limited to isolated clinical tools; they now participate in scheduling, documentation, communication, referral management, remote follow-up, workload distribution, and the generation of operational data for decision-making. Recent studies in telehealth and EHR-enabled care have shown that digital interventions may support referral efficiency, care continuity, access, and service coordination in primary care settings [1–4].

Primary care is a particularly important setting for evaluating digital health management because it concentrates high-volume interactions, longitudinal follow-up, population health activities, and coordination across levels of care. Health systems increasingly rely on primary care to address aging populations, multimorbidity, workforce constraints, and the growing demand for timely access. In this context, digital health technologies are expected to support more adaptive and data-informed management models. Telemedicine and mHealth can extend access and support remote monitoring, especially in rural or mobility-constrained populations [5–8]. EHRs, patient portals, and interoperable platforms can improve documentation traceability and communication, although they may also alter professional workload and inbox burden [4,9,10]. AI/ML applications, in turn, are being explored for risk prediction, screening, demand forecasting, and resource planning [11–13].

Despite this expansion, the evidence on digital health management outcomes in primary care remains fragmented. Many studies focus on clinical effectiveness, adoption, usability, or patient experience, whereas fewer provide standardized measures of operational performance. Indicators such as waiting times, administrative efficiency, resource utilization, operating costs, continuity of care, and patient or professional satisfaction are often defined differently across studies. This heterogeneity limits direct comparison between interventions and reduces the feasibility of pooled inferential synthesis. It also creates uncertainty for policymakers and service managers seeking to determine which digital technologies produce measurable organizational value across health-system contexts.

A further challenge is that digital health implementation depends on the governance conditions under which data are produced, exchanged, and reused. Privacy regulations, interoperability standards, procurement capacity, technical infrastructure, digital literacy, and workforce readiness vary substantially across countries and regions. These differences are especially relevant in resource-limited or infrastructure-constrained settings, where connectivity, device access, documentation quality, and technical support may shape whether digital tools improve or worsen service performance. Telemedicine studies have increasingly highlighted equity concerns, including differential access by language preference, rurality, digital exclusion, and social context [5,8,14–16]. Broader digital health reviews also show that implementation in resource-constrained health systems is shaped by governance capacity, infrastructure readiness, financing, and workforce preparedness [17]. Therefore, digital maturity in primary care cannot be evaluated only as technology adoption; it must also be examined as a governance, infrastructure, and implementation problem.

AI/ML and NLP introduce additional methodological and ethical concerns. Predictive analytics and text-processing tools may support workload prioritization, documentation, triage, and resource allocation. Still, they also depend on the quality and representativeness of the data used for development and deployment. Data imbalance, missingness, language variation, underrepresentation of vulnerable populations, and unequal access to digital services may produce biased or poorly generalizable models. These risks are particularly important when algorithmic outputs influence administrative prioritization, demand forecasting, or allocation of care resources. The emerging view of medical data as a strategic institutional asset emphasizes that data value depends not only on volume, but also on governance, quality, interoperability, accountability, and ethical safeguards [18].

Previous reviews have examined telemedicine, digital health, or AI applications in healthcare, but many have emphasized clinical outcomes, access, or disease-specific effectiveness rather than management indicators in primary care [19–21]. Less attention has been given to how different digital technologies relate to measurable organizational outcomes, how consistently those outcomes are reported, and whether the evidence base supports inferential pooling or only descriptive synthesis. This distinction is important because health-service managers require evidence on operational consequences, not only on clinical acceptability or technical feasibility.

This systematic review addresses that gap by synthesizing evidence on digital health technologies and management outcomes in primary care. The review focuses on EHR/interoperability, telemedicine/mHealth, AI/ML or predictive analytics, NLP/text processing, and mixed digital health interventions. The outcomes of interest include waiting times, administrative efficiency, resource utilization, operating costs, quality-related management indicators, continuity of care, and patient or professional satisfaction. The following question guided the review:

What is the existing evidence on the association between digital health technologies, including EHR/interoperability, telemedicine/mHealth, AI/ML, and NLP, and measurable management outcomes in primary care, compared with routine care, non-digital management models, alternative digital interventions, or pre-implementation baselines?

By focusing on management outcomes, this review aims to clarify the structure, scope, and reporting quality of the evidence base. It also examines whether the available literature supports formal inferential synthesis or is better characterized through descriptive quantitative synthesis, temporal sensitivity analysis, and validated subsample risk-of-bias assessment.

## 2. Materials and methods

### 2.1. Review design and eligibility framework

This systematic review was conducted following the Preferred Reporting Items for Systematic Reviews and Meta-Analyses (PRISMA 2020) guideline to ensure transparency in the identification, screening, eligibility assessment, and synthesis of evidence [22]. The review was designed to examine the relationship between digital health technologies and measurable management outcomes in primary care. Because the eligible literature included heterogeneous study designs, intervention types, outcome definitions, measurement scales, and statistical reporting formats, the synthesis was planned as a systematic review with descriptive quantitative synthesis rather than as a formal meta-analysis.

The review question was structured using the Population, Intervention, Comparator, Outcomes (PICO) framework. The population of interest comprised primary care services, community health services, and health-system settings where primary care management processes were evaluated. The interventions included digital health technologies applied to service management, including EHR/EMR, interoperable health information systems, telemedicine, mHealth, AI, ML, predictive analytics, and NLP [23–25]. Comparators included routine care, non-digital management models, alternative digital interventions, or pre-implementation baselines when no parallel comparator was available. Outcomes were restricted to management-related indicators, including waiting times, administrative efficiency, resource utilization, operating costs, care quality, continuity of care, and patient or professional satisfaction [26–28].

Eligible studies were peer-reviewed original articles published in English between 2018 and 2025. Randomized studies, quasi-experimental studies, observational studies, implementation studies, pilot studies, and mixed-methods studies were eligible if they reported at least one management-related outcome associated with a digital health intervention in primary care or community-based services. Studies were excluded when they were secondary reviews, protocols without results, conference abstracts, editorials, commentaries, letters, purely technical algorithm-development studies without health-service evaluation, simulations without validation in a healthcare setting, or studies reporting exclusively clinical outcomes without an organizational or management link. Table 1 summarizes the eligibility framework.

**Table 1:** Eligibility criteria applied in the systematic review.

Category	Inclusion criteria	Exclusion criteria
Study type	Original peer-reviewed studies, including randomized, quasi-experimental, observational, implementation, pilot, and mixed-methods studies with management-related outcomes.	Secondary reviews, protocols without results, editorials, letters, commentaries, conference abstracts, and reports without explicit methodology.
Setting	Primary care, community care, outpatient management, or health-system contexts directly linked to primary care service delivery.	Hospital-only studies without primary care relevance or studies conducted exclusively in laboratory, simulation, or software-testing environments.
Intervention	EHR/EMR, interoperability, telemedicine, mHealth, AI/ML, predictive analytics, NLP, or digital health systems used for service management.	Purely clinical interventions or algorithm-development studies without evaluation in a healthcare management context.
Outcomes	Waiting times, administrative efficiency, resource utilization, operating costs, quality of care, continuity of care, and patient or professional satisfaction.	Clinical outcomes with no organizational link, such as drug efficacy, biomarkers, or diagnostic accuracy alone.
Publication features	English-language peer-reviewed articles published from 2018 to 2025 with sufficient title, abstract, and bibliographic metadata for screening.	Studies published before 2018, non-English records, retracted publications, or records with insufficient metadata for eligibility assessment.

**Notes:** EHR/EMR = electronic health/medical records; AI = artificial intelligence; ML = machine learning; NLP = natural language processing.

### 2.2. Information sources and search strategy

The search strategy was designed to retrieve biomedical, health-management, and applied informatics literature addressing digital health technologies and management outcomes in primary care. Searches covered records published from January 1, 2018, to October 7, 2025, corresponding to the final documented export and corpus consolidation date. Searches were conducted in PubMed/MEDLINE, Web of Science, Scopus, CINAHL (EBSCO), and IEEE Xplore. These sources were selected to cover clinical medicine, public health, nursing and community care, health services management, multidisciplinary research, engineering, and applied computer science. The selected databases correspond to the sources used in the final corpus construction and PRISMA flow.

Search terms were organized into three conceptual blocks derived from the PICO framework: (i) primary care or community health services; (ii) digital health technologies, including EHR/EMR, telemedicine, mHealth, AI/ML, NLP, and interoperability; and (iii) management outcomes, including efficiency, waiting time, resource utilization, cost, quality improvement, access, and satisfaction. Controlled vocabulary was used when available, including MeSH in PubMed/MEDLINE and CINAHL Headings in CINAHL. Free-text terms were searched in titles, abstracts, keywords, or topic fields, depending on the structure of each database. Boolean operators were used to combine terms within and across blocks, and the PubMed/MEDLINE strategy served as the reference structure for adapting syntax to other databases. Table 2 summarizes the information sources, search fields, and contribution of each database to the review.

**Table 2:** Information sources and search-strategy adaptation.

Database	Coverage	Search fields or vocabulary	Contribution to the review
PubMed / MEDLINE	Biomedical and public health literature	MeSH terms and title/abstract fields.	Core biomedical source for primary care, health services, clinical management, and digital health research.
Web of Science	International multidisciplinary literature	Topic fields combining title, abstract, author keywords, and Keywords Plus.	Citation-indexed multidisciplinary source covering health, management, public health, and social science literature.
Scopus	International multidisciplinary literature	Title, abstract, and keyword fields.	Broad interdisciplinary source for health technology implementation, digital health, and service-management research.
CINAHL (EBSCO)	Nursing, community health, and care management	CINAHL Headings and free-text fields.	Source for nursing, primary care, community health, care coordination, and care-management studies.
IEEE Xplore	Engineering, computer science, and applied informatics	Metadata and abstract fields using free-text search terms.	Technical source for health information systems, AI/ML, NLP, interoperability, and applied digital health systems.

The reference PubMed/MEDLINE string combined MeSH terms and title/abstract terms. The complete database-specific search strategies, including Boolean operators and field tags, are provided in the supplementary search-strategy appendix. The reference structure is shown in Listing 1.

**Listing 1. Reference PubMed/MEDLINE search string**

((“Primary Health Care”[MeSH] OR “primary care”[tiab] OR “community health”[tiab]

OR “health services management”[tiab] OR “health administration”[tiab])

AND

(“Health Information Systems”[MeSH] OR “Electronic Health Records”[MeSH]

OR “Artificial Intelligence”[MeSH] OR “Natural Language Processing”[MeSH]

OR “Telemedicine”[MeSH] OR “information technology”[tiab]

OR “electronic health record”[tiab] OR EHR[tiab] OR EMR[tiab]

OR “machine learning”[tiab] OR “natural language processing”[tiab]

OR NLP[tiab] OR “digital health”[tiab] OR mHealth[tiab]

OR interoperability[tiab])

AND

(“Efficiency, Organizational”[MeSH] OR “Quality of Health Care”[MeSH]

OR “Patient Satisfaction”[MeSH] OR “Health Services Accessibility”[MeSH]

OR efficiency[tiab] OR productivity[tiab] OR “waiting time”[tiab]

OR “resource utilization”[tiab] OR cost[tiab] OR “quality improvement”[tiab]

OR satisfaction[tiab]))

AND (“2018/01/01”[Date - Publication]: “2025/10/07”[Date - Publication])

### 2.3. Record management, screening, and final eligibility audit

Records retrieved from each database were exported in RIS, BibTeX, or CSV format, depending on platform availability, and consolidated into a structured bibliographic repository. The initial search retrieved 12,160 records across PubMed/MEDLINE, Web of Science, Scopus, CINAHL (EBSCO), and IEEE Xplore. After database-level filters and export restrictions were applied, 2,444 records were retained for integration. Duplicate detection was performed using exact and near-exact matching of title, DOI, year, and first author. Automated matching was complemented by manual verification of borderline cases to avoid removing distinct publications with similar titles or retaining duplicate database exports, in line with reproducibility principles recommended for systematic review workflows [22].

Screening was conducted in sequential stages following the PRISMA 2020 logic [22]. Title and abstract screening were used to identify records potentially related to digital health technologies, primary care, or community care, and management outcomes. Records that did not address a digital health intervention, did not involve a relevant health-service setting, or did not report management-related information were excluded. This thematic screening stage retained 1,034 records. Eligibility assessment then focused on study type, availability of empirical results, presence of extractable management indicators, and completeness of bibliographic and abstract-level metadata. This process produced a candidate analytical corpus of 256 records for the initial descriptive synthesis.

A final eligibility audit was conducted to verify the consistency, reporting completeness, and analytical suitability of the candidate corpus before synthesis. The 256-record analytical corpus was rechecked for abstract availability, empirical result status, secondary-review status, and extractability of management-related information. This audit excluded 84 records: 35 for missing abstracts, 34 for being protocols or no-results records, 10 for being secondary reviews, and 5 for meeting multiple exclusion criteria. Pilot, feasibility, or setting-context studies were retained when they reported management-relevant information, but they were flagged for cautious interpretation in the synthesis. This audit resulted in a final cleaned corpus of 172 studies.

The screening and final eligibility audit were implemented using reproducible Python scripts and structured CSV files. Automated text processing was used to standardize metadata, identify candidate technology and outcome categories, and flag potential reasons for exclusion. However, final inclusion decisions were based on the predefined eligibility framework and documented audit rules rather than solely on an automated classifier. No automated risk-of-bias or study-quality score was used as a substitute for a validated methodological appraisal tool. This distinction was maintained to align the review workflow with transparent reporting and reproducible evidence-synthesis practices [22]. The resulting PRISMA-style selection flow is presented in Fig 1.

**Fig 1:**
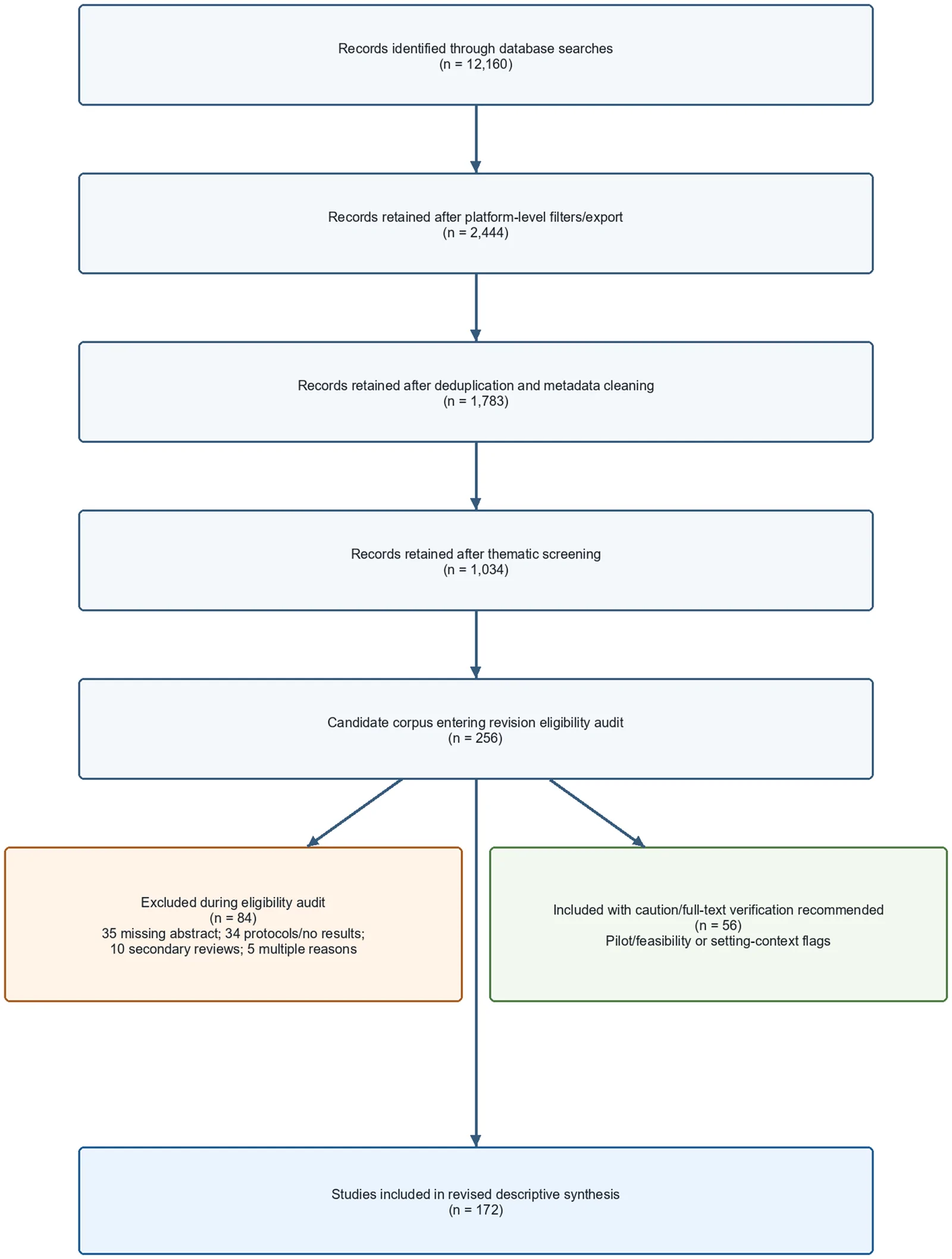
PRISMA-style selection flow for the final cleaned corpus. The diagram summarizes record identification, platform-level filtering, deduplication and metadata cleaning, thematic screening, final eligibility audit, and inclusion in the descriptive quantitative synthesis.

### 2.4. Data extraction and classification of technologies and outcomes

Data extraction was conducted using a structured template designed around the PICO framework and the review objectives. The template was used to capture bibliographic metadata, year of publication, care setting, study design, population or user group, digital health technology, management indicators, statistical reporting language, and, when available, the direction of reported management findings. Extracted fields were standardized before synthesis to reduce variation in terminology across databases and article abstracts, in line with PRISMA 2020 principles for transparent data collection and reporting [22]. Missing or unavailable information was coded as not reported (NR) to preserve transparency and avoid imputing study characteristics that were not explicitly available.

Technological interventions were classified into five categories: telemedicine/mHealth, EHR/interoperability, AI/ML or predictive analytics, NLP/text processing, and mixed or other digital health. These categories were selected to reflect the main digital health domains identified in the eligible corpus while maintaining consistency with the technologies specified in the eligibility framework. Two coding layers were used. First, each study was assigned to a single primary technology category to support mutually exclusive characterization of the corpus. Second, non-mutually exclusive technology mentions were retained for analyses linking technology categories to management indicators, because many interventions combined remote care, electronic records, interoperability, or data-driven components.

Primary technology coding followed a hierarchical decision rule. Studies explicitly focused on NLP or text-processing applications were coded as NLP/text processing; studies centered on predictive models, ML, or algorithm-supported decision tools were coded as AI/ML or predictive analytics; studies focused on EHRs, information exchange, portals, or interoperability infrastructure were coded as EHR/interoperability; studies centered on remote consultation, telehealth, mobile health, or digital follow-up were coded as telemedicine/mHealth. Studies that could not be assigned to a single dominant technology, or that described broad digital transformation without a clearly dominant component, were coded as mixed or as other digital health.

Management indicators were classified into five outcome groups: waiting times, administrative efficiency, resource utilization, operating costs, and patient or professional satisfaction. Indicator coding was based on abstract- and metadata-level evidence, with additional flags for explicit language of statistical significance when available. The coding framework prioritized management and organizational outcomes; clinical outcomes were recorded only when they were directly connected to service delivery, workflow, resource allocation, or administrative performance. Statistical reporting was coded descriptively as present when abstracts or available metadata explicitly reported terms such as statistically significant, confidence interval, odds ratio, mean difference, regression estimate, or p value linked to a management outcome. Table 3 summarizes the extracted variables and their role in the descriptive synthesis.

**Table 3:** Variables extracted and coding purpose.

Domain	Extracted variables	Purpose in synthesis
Bibliographic metadata	Title, authors, year, DOI, source database, abstract availability.	Deduplication, traceability, and eligibility assessment.
Study characteristics	Study design, care setting, population or user group, implementation context.	Description of evidence base and identification of applicability to primary care management.
Technology coding	Primary technology category and non-mutually exclusive technology mentions.	Characterization of digital health interventions and technology-indicator matrices.
Management indicators	Waiting times, administrative efficiency, resource utilization, operating costs, and patient/professional satisfaction.	Descriptive quantitative synthesis of management outcomes.
Reporting features	Statistical significance language, availability of quantitative indicators, and caution flags.	Assessment of reporting completeness and suitability for descriptive synthesis.

A targeted full-text verification step was applied to the records contributing directly to the management-outcome synthesis. The verification set comprised the 53 records initially coded as reporting at least one management indicator. For each record, the full text was matched to the bibliographic record, and the presence of the coded management indicator, study design information, statistical-reporting language, and source location within the article were checked against the PDF or full-text source. Records identified during this verification step as correction notices, metadata exports, commentaries, secondary reviews, or non-empirical texts were flagged for recoding or exclusion from the management-outcome synthesis rather than retained on the basis of abstract-level coding alone. The verified set was used as the basis for the management-indicator counts reported in the Results, while records with incomplete or non-confirmatory full-text evidence were interpreted only descriptively and were not used to support inferential or causal claims. The record-level verification matrix and codebook are provided in S1 Data. This verification step was used to reduce the risk that outcome classification depended only on titles, abstracts, or database metadata.

### 2.5. Descriptive quantitative synthesis and temporal sensitivity analysis

The evidence was synthesized using descriptive quantitative methods, following the PRISMA 2020 emphasis on transparent reporting of synthesis decisions [22]. Frequencies and percentages were calculated for publication year, primary technology category, non-mutually exclusive technology mentions, management indicators, and technology-by-indicator relationships. The primary unit of analysis was the study record. For mutually exclusive summaries, each study contributed once to one dominant technology category. For technology-by-indicator matrices, studies could contribute to more than one technology category when the intervention included multiple digital components.

A formal meta-analysis was not performed because the included studies differed substantially in design, intervention content, comparator structure, outcome definitions, measurement scales, follow-up periods, and statistical reporting. In addition, many records reported management outcomes descriptively or used non-comparable indicators without extractable effect estimates. Therefore, pooled effect sizes, confidence intervals, and heterogeneity statistics such as I^2^, $\tau^{2}$, and Cochran’s Q were not calculated. The synthesis reports distributions, normalized frequencies, and technology-indicator patterns rather than random-effects meta-analytic estimates.

The technology-by-indicator analysis used non-mutually exclusive coding to preserve the layered structure of digital health interventions. For example, studies combining telemedicine platforms with EHR access, patient portals, or interoperable information systems could contribute to more than one technology category in the indicator matrix, while still contributing only once to the mutually exclusive primary technology summary. This distinction enabled the synthesis to separate the corpus’s general composition from the more complex relationships between digital components and management indicators.

Management indicators were summarized as record-level mentions and, where available, as records reporting explicit statistical significance language. Statistical significance was not interpreted as a pooled effect estimate; it was used only as a reporting-completeness marker indicating whether a study provided inferential evidence for a management outcome. This approach was selected to avoid combining non-equivalent outcomes or overstating causal effects in a literature base characterized by heterogeneous designs and variable reporting quality.

A temporal sensitivity analysis was conducted to evaluate whether the post-2021 focus of the analytical corpus could materially alter the descriptive technology profile. A comparable corpus covering 2018–2025 was cleaned using the same exclusion logic applied in the final eligibility audit, including removal of records with missing abstracts, protocols, no-results status, or secondary review status. Records from 2018–2021 and 2022–2025 were then compared using technology-category mentions per 100 records.

The temporal sensitivity analysis also included management-outcome categories. The outcome-level sensitivity analysis compared the frequency of explicitly coded management indicators in 2018–2021 and 2022–2025 using the same normalized metric of mentions per 100 records. This analysis was intended to evaluate whether the post-2021 analytical focus changed the distribution of management outcomes, not to estimate intervention effects or temporal trends in effectiveness.

Mentions per 100 records were calculated as:


$$\begin{array}{c} \text{Mentions per 100 records}=\left(\frac{\text{Number of records mentioning the technology category}}{\text{Total records in the period}}\right)\times 100 \end{array}$$

This normalization allowed comparison between periods with different corpus sizes and provided a descriptive check of whether inclusion of earlier studies would change the main technology distribution. The sensitivity analysis was not intended to estimate intervention effects over time; it was used to assess the stability of the corpus structure and the potential influence of temporal narrowing on the descriptive synthesis.

### 2.6. Risk-of-bias assessment and reproducibility

Methodological quality was assessed using a validated subsample risk-of-bias check based on the Joanna Briggs Institute (JBI) checklist domains [29]. A stratified subsample of 40 studies was selected from the final cleaned corpus to represent the main primary technology categories: telemedicine/mHealth (n = 16), EHR/interoperability (n = 8), AI/ML or predictive analytics (n = 8), NLP/text processing (n = 2), and mixed or other digital health (n = 6). Stratification was used to avoid restricting the assessment to the dominant telemedicine/mHealth category and to ensure that less frequent technology groups were represented in the methodological appraisal.

JBI checklist domains were selected because the final corpus included heterogeneous study designs, including observational, quasi-experimental, implementation, pilot, and mixed-methods studies. Although RoB 2 and ROBINS-I are widely used for randomized and non-randomized intervention studies, respectively [30,31], the diversity of designs and reporting formats in this review required a flexible appraisal structure applicable across multiple empirical designs. The assessment focused on domains relevant to non-randomized and implementation-oriented evidence, including clarity of study design, appropriateness of participant or setting selection, description of the intervention or exposure, validity of outcome measurement, identification of potential confounding, appropriateness of statistical analysis, and completeness of reporting.

The JBI subsample assessment was conducted using abstract-level and metadata-level information. Studies were classified as low preliminary risk when the available information described the design, setting, intervention, outcome measurement, and analysis with sufficient clarity. Studies were classified as having a moderate preliminary risk when key domains were partially reported, and no major methodological concerns were evident from the available information. Studies were classified as high/unclear preliminary risk when essential information on design, sampling, comparator, measurement, confounding, or statistical analysis could not be verified from the abstract-level record. Records with incomplete reporting were flagged as requiring full-text confirmation rather than being interpreted as definitively high risk.

The preliminary JBI classifications were recorded in structured extraction files and summarized by overall risk category and by primary technology category. This approach allowed the synthesis to report the distribution of low, moderate, and high/unclear preliminary risk within a validated subsample while preserving transparency about the limits of abstract-level appraisal. The JBI check was used to contextualize the descriptive synthesis and to evaluate whether the final corpus showed major reporting-quality imbalances across technology categories.

The JBI subsample check was not interpreted as a complete study-level risk-of-bias assessment for all 172 included records. Instead, it was retained as a validated, stratified appraisal used to contextualize reporting quality across technology categories. The JBI subsample was therefore not used as evidence that the entire corpus had low or moderate risk of bias. The main safeguard for the outcome synthesis was the targeted full-text verification of records contributing directly to management indicators.

All corpus cleaning, eligibility audit, classification, descriptive synthesis, temporal sensitivity, JBI subsample, table generation, and figure generation steps were implemented in Python using structured CSV inputs and outputs. Scripts were organized to preserve the traceability of each analytical stage, including the original cleaned corpus, final eligibility decisions, exclusion reasons, included-with-caution flags, technology coding, management-indicator coding, temporal sensitivity outputs, and JBI subsample summaries. The manuscript reports the analytical outputs generated from these scripts, and the underlying datasets, decision logs, and scripts are provided as supplementary materials to support verification and reproducibility in accordance with transparent reporting principles for systematic reviews [22].

## 3. Results

### 3.1. Final corpus and eligibility outcome

The final eligibility audit reduced the candidate analytical corpus from 256 records to 172 studies included in the descriptive quantitative synthesis. This refinement was performed to strengthen the internal consistency of the evidence base by reassessing eligibility, reporting completeness, and suitability for descriptive quantitative synthesis. The final corpus, therefore, represents the subset of records with sufficient bibliographic, abstract-level, and outcome-related information to support the results reported in the following sections.

A total of 84 records were excluded from the final core corpus because the available metadata indicated the absence of an abstract (n = 35), protocol or no-results status (n = 34), secondary review status (n = 10), or multiple exclusion reasons (n = 5). These exclusions primarily removed records that could not support direct extraction of management-related evidence or that did not report empirical findings. An additional 56 records were retained with caution because they included pilot, feasibility, or setting-context flags but still reported management-relevant information. These studies were preserved in the descriptive synthesis but interpreted conservatively. Table 4 summarizes the final eligibility outcome.

**Table 4:** Final corpus eligibility outcome.

Eligibility outcome	Records (n)	Percentage and denominator	Main interpretation
Candidate analytical corpus	256	100.0% of candidate corpus	Records entering the final eligibility audit
Included in final cleaned corpus	172	67.2% of candidate corpus	Core descriptive synthesis set
Excluded from final cleaned corpus	84	32.8% of candidate corpus	Missing abstract, protocol/no-results, secondary review status, or multiple exclusion reasons
Included with caution	56	32.6% of final cleaned corpus	Subset retained within the 172 studies but flagged for cautious interpretation

The retained corpus preserves the post-2021 analytical focus while applying a stricter eligibility audit to reduce the influence of incomplete records, non-empirical publications, and studies without extractable management outcomes. Subsequent results are therefore reported using the final cleaned corpus (n = 172), with sensitivity analyses used to evaluate whether the exclusion of earlier records could materially affect the interpretation of the evidence base.

### 3.2. Characteristics of the final cleaned corpus

The final cleaned corpus comprised 172 studies published between 2022 and 2025. The annual distribution was balanced across the study window, with 44 studies in 2022, 44 in 2023, 43 in 2024, and 41 in 2025. This distribution indicates that the final corpus was not concentrated in a single publication year and that the post-2021 evidence base was relatively stable across the period analyzed.

Technology categories were first summarized using a mutually exclusive primary classification. Each study was assigned to a single dominant category to avoid double-counting in the overall characterization of the corpus. Under this classification, telemedicine/mHealth represented the largest group (n = 98; 57.0%), followed by EHR/interoperability (n = 35; 20.3%) and AI/ML or predictive analytics (n = 29; 16.9%). NLP/text-processing studies were uncommon (n = 2; 1.2%), and mixed or other digital health interventions represented a smaller residual group (n = 8; 4.7%). Table 5 summarizes this mutually exclusive distribution.

**Table 5:** Primary technology category in the final cleaned corpus (n = 172).

Primary technology category	Studies (n)	Percentage
Telemedicine/mHealth	98	57.0%
EHR/Interoperability	35	20.3%
AI/ML/Predictive analytics	29	16.9%
NLP/Text processing	2	1.2%
Mixed/other digital health	8	4.7%

The distribution shows that the final evidence base was primarily organized around service-delivery technologies, particularly telemedicine and mHealth. This pattern is consistent with studies evaluating telehealth capacity, equity-oriented telehealth adaptation, community health worker-facilitated telehealth, and satisfaction with rural telemedicine in primary care contexts [1,5–7,32]. EHR/interoperability studies formed the second-largest group, reflecting the continued relevance of information infrastructure, EHR-integrated decision support, workflow tools, and patient portal communication in primary care management [4,9,33,34]. AI/ML and predictive analytics were present but less frequent, consistent with studies applying ML, AI-supported decision tools, risk prediction, and screening algorithms to primary care or outpatient management tasks [11,13,28,35]. NLP/text-processing studies were rare, although recent work on ambient documentation and generative AI for clinical documentation illustrates the emerging relevance of text-processing tools for administrative and documentation workflows [36,37].

The year-by-technology matrix in Fig 2A shows that telemedicine/mHealth remained the dominant category throughout the four years. However, its absolute count decreased slightly from 27 studies in 2022–24 in 2025. EHR/interoperability remained comparatively stable, with 8–10 studies per year, while AI/ML and predictive analytics showed a stable annual presence of 6–8 studies. NLP/text processing appeared only in 2024 and 2025, reinforcing its limited representation within the final corpus.

**Fig 2:**
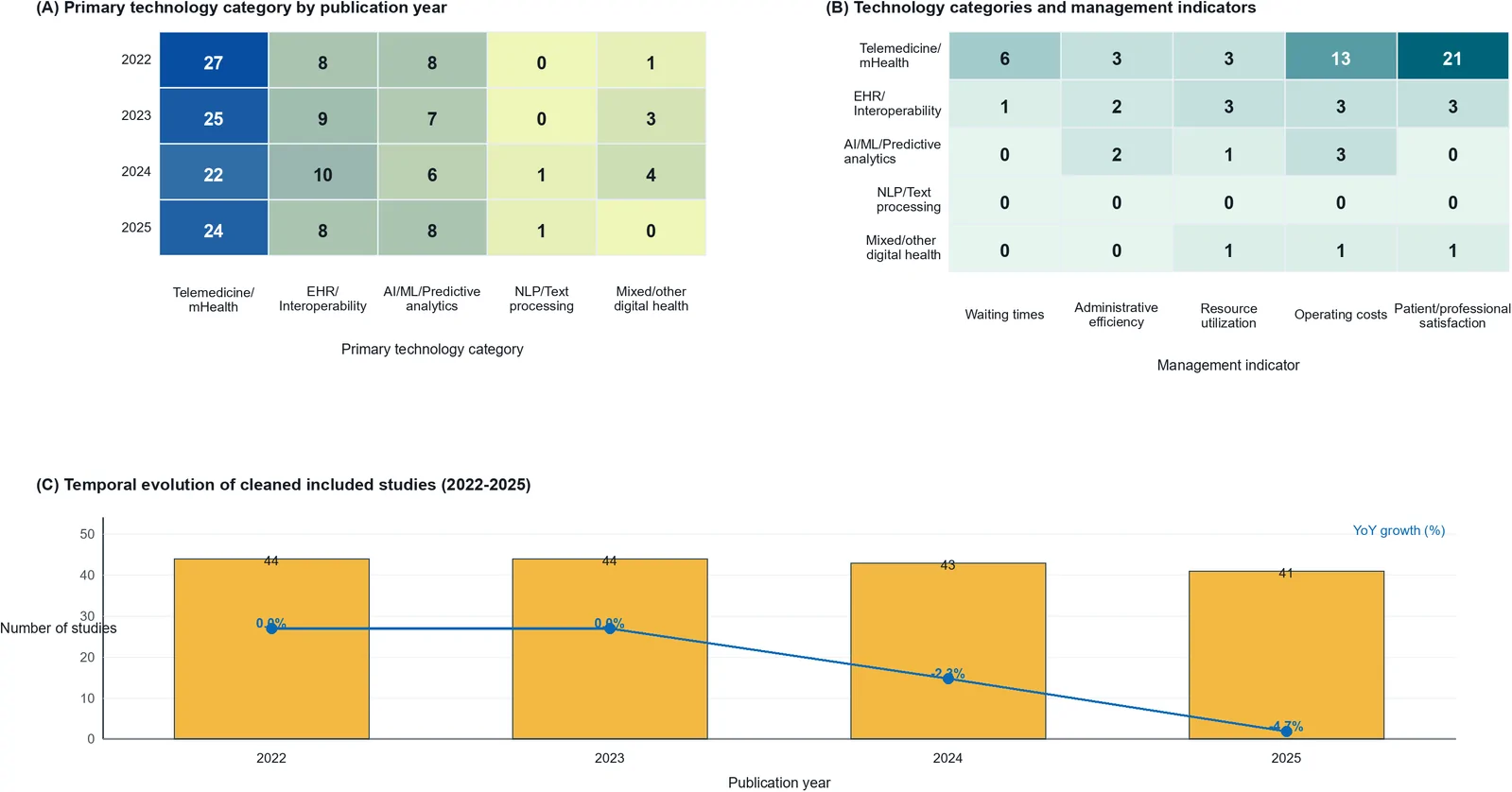
Descriptive profile of the final cleaned corpus. Panel A shows primary technology categories by publication year.Panel B shows non-mutually exclusive relationships between technology categories and management indicators. Panel C shows the temporal distribution of included studies from 2022 to 2025.

Because many digital health interventions combine more than one technological component, a secondary non-mutually exclusive classification was retained for the indicator-by-technology synthesis. Under this broader coding, telemedicine/mHealth appeared in 109 studies (63.4%), EHR/interoperability in 39 studies (22.7%), AI/ML or predictive analytics in 31 studies (18.0%), mixed or other digital health in 8 studies (4.7%), and NLP/text processing in 2 studies (1.2%). The difference between the primary and non-mutually exclusive counts was most evident for telemedicine/mHealth and EHR/interoperability, reflecting studies in which remote care models were linked to electronic records, patient portals, or other information-system components.

This dual characterization separates the corpus’s overall structure from the more complex relationships between technologies and management indicators. The mutually exclusive classification provides a clear denominator for describing the included studies. In contrast, the non-mutually exclusive classification preserves the layered nature of digital health interventions when examining their association with operational outcomes.

### 3.3. Descriptive synthesis of management indicators

Management indicators were reported heterogeneously across the final corpus, both in terminology and in the level of quantitative detail available for extraction. The most frequently identified indicators were patient/professional satisfaction (n = 24), operating costs (n = 20), resource utilization (n = 8), waiting times (n = 7), and administrative efficiency (n = 7). This distribution shows that the evidence base emphasized experiential and economic outcomes more frequently than direct measures of operational flow or administrative workload. Studies of telehealth resource utilization, patient satisfaction, video-visit scheduling, implementation costs, and quality or access outcomes illustrate this emphasis on service experience and economic dimensions [38–43].

Explicit reporting of statistical significance was uncommon across indicator groups. Significant language was identified in two studies reporting resource utilization, one study reporting operating costs, and one study reporting patient/professional satisfaction. No explicit reporting of statistical significance was detected for waiting times or administrative efficiency in the cleaned indicator matrix. This limited availability of comparable inferential outputs supports presenting the evidence as a descriptive quantitative synthesis rather than as a pooled meta-analysis. Table 6 summarizes the management indicators identified in the final cleaned corpus and the proportion of studies reporting statistical significance.

**Table 6:** Management indicators identified in the final cleaned corpus.

Management indicator	Studies mentioning indicator	Studies reporting statistical significance	Percentage with significance reporting
Waiting times	7	0	0.0%
Administrative efficiency	7	0	0.0%
Resource utilization	8	2	25.0%
Operating costs	20	1	5.0%
Patient/professional satisfaction	24	1	4.2%

The technology-by-indicator matrix showed that telemedicine/mHealth accounted for most indicator mentions, particularly patient/professional satisfaction (n = 21) and operating costs (n = 13). Telemedicine/mHealth was also the category most frequently linked to waiting times (n = 6), although this indicator remained less commonly reported overall. This pattern suggests that remote care interventions were most often evaluated through patient-facing, access-related, or service-use outcomes rather than through standardized administrative efficiency measures [39–42].

EHR/interoperability showed a more evenly distributed profile, with mentions across waiting times (n = 1), administrative efficiency (n = 2), resource utilization (n = 3), operating costs (n = 3), and patient/professional satisfaction (n = 3). This distribution is consistent with the operational role of record systems and interoperability tools, which tend to affect multiple management domains rather than a single outcome category. Studies on EHR data reuse, EHR efficiency tools, interprofessional EHR-based care, and ambulatory EHR workload illustrate how these systems can shape administrative burden, coordination, and productivity-related outcomes [9,10,44–46]. AI/ML and predictive analytics were mainly associated with administrative efficiency (n = 2), resource utilization (n = 1), and operating costs (n = 3), with no satisfaction-related mentions detected in the cleaned indicator matrix. Examples of AI-enabled efficiency, quality-measure prediction, and machine-learning screening illustrate the emerging management orientation of these tools [13,47,48]. NLP/text processing did not contribute explicit management-indicator mentions in this matrix. Fig 3A summarizes the frequency of management indicators in the final corpus, while Fig 3B shows their distribution by technology category using non-mutually exclusive coding.

**Fig 3:**
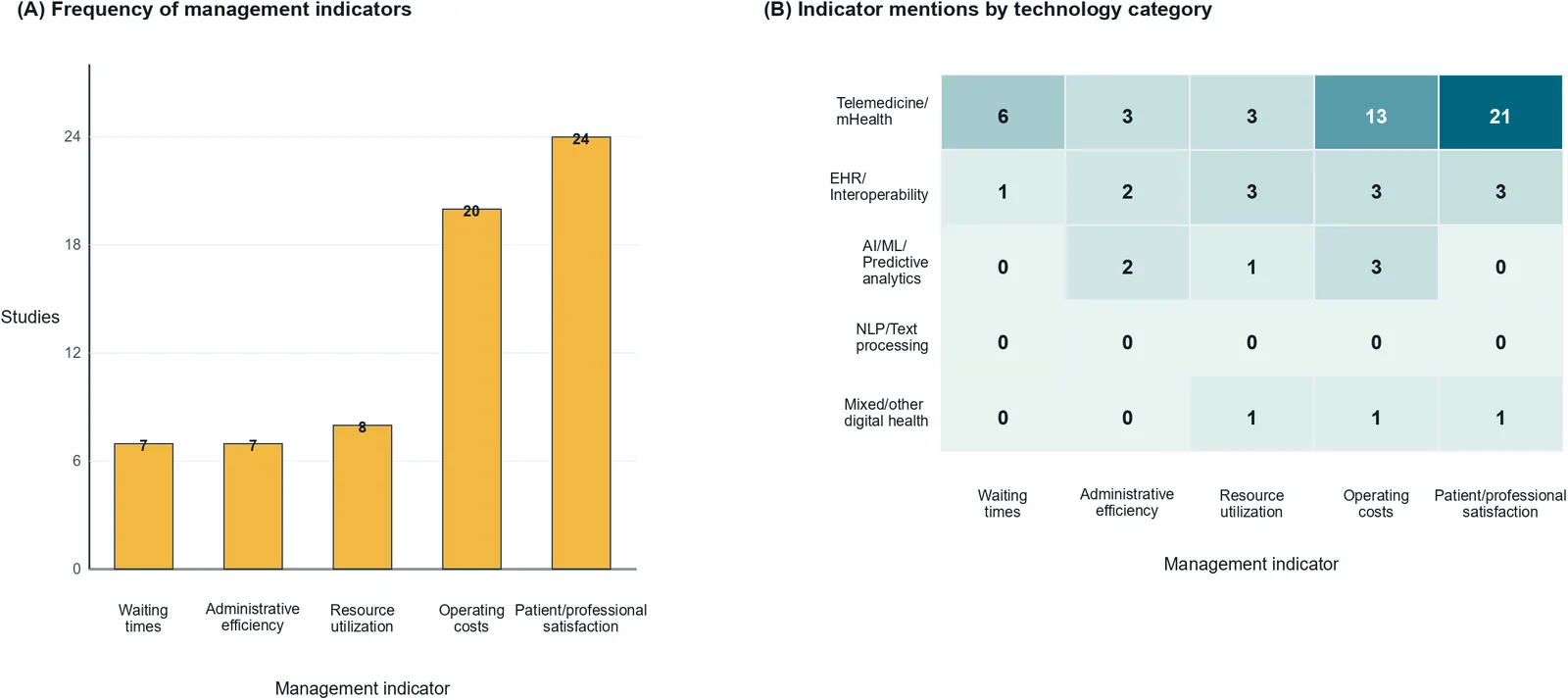
Management indicators in the final cleaned corpus. Panel A shows the frequency of management indicators. Panel B shows indicator mentions by technology category using non-mutually exclusive coding.

The final corpus provided sufficient information to describe the frequency and distribution of management indicators across technology categories, but not enough standardized effect estimates to support formal pooling. For this reason, the synthesis focuses on indicator presence, technology-outcome alignment, and the availability of statistical reporting rather than pooled effect sizes or heterogeneity statistics.

### 3.4. Temporal sensitivity analysis

A temporal sensitivity analysis was conducted to examine whether the post-2021 analytical focus of the final corpus could have materially changed the distribution of technology categories. For this analysis, a cleaned comparable corpus covering 2018–2025 was constructed using the same exclusion logic applied to the final corpus, including removal of records with missing abstracts, protocol, or no-results status, and secondary review status. After this harmonized cleaning process, 1,138 of 1,685 comparable records remained: 585 from 2018–2021 and 553 from 2022–2025.

Technology-category frequencies were normalized to mentions per 100 records to allow comparison across periods with different corpus sizes. This normalization was necessary because the sensitivity analysis used a broader comparable corpus rather than the final synthesis set. The objective was not to re-estimate pooled effects, but to assess whether inclusion of pre-2022 records would substantially alter the descriptive structure of the evidence base.

The broad distribution of technology categories was similar across periods for the two largest infrastructure and service-delivery domains. EHR/interoperability remained frequent in both windows, increasing modestly from 40.3 mentions per 100 records in 2018–2021 to 43.6 mentions per 100 records in 2022–2025. Telemedicine/mHealth also remained prominent, increasing from 25.3 to 31.8 mentions per 100 records. This pattern is consistent with the broader expansion and normalization of telemedicine before, during, and after the COVID-19 period, particularly in primary care and community health settings [49–51]. These findings indicate that the main technology domains identified in the final corpus were already present in the earlier literature and were not artifacts of the 2022–2025 restriction.

The largest proportional increase was observed for AI/ML and predictive analytics, which rose from 6.2 to 15.6 mentions per 100 records. This pattern suggests a stronger representation of data-driven and predictive applications in the more recent period, consistent with recent work that positions AI as a tool to support primary care performance and management functions [52]. NLP/text processing remained uncommon in both windows, with only a small increase from 1.4 to 1.8 mentions per 100 records. In contrast, mixed or other digital health decreased from 32.5 to 15.7 mentions per 100 records, suggesting that more recent records were more likely to be classifiable into specific technology categories rather than broad digital health labels. This shift is compatible with the increasing specification of digital health applications and integrated care models in recent primary care literature [53,54]. Table 7 summarizes the normalized temporal sensitivity analysis by technology category.

**Table 7:** Temporal sensitivity analysis by technology category.

Technology category	2018–2021 mentions per 100 records	2022–2025 mentions per 100 records	Absolute difference
Telemedicine/mHealth	25.3	31.8	+6.5
EHR/Interoperability	40.3	43.6	+3.3
AI/ML/Predictive analytics	6.2	15.6	+9.4
NLP/Text processing	1.4	1.8	+0.4
Mixed/other digital health	32.5	15.7	-16.8

Fig 4 summarizes the normalized temporal comparison. The sensitivity analysis did not show a reversal of the main technology profile when the earlier period was considered. Instead, it showed continuity in EHR/interoperability and telemedicine/mHealth, greater visibility of AI/ML in the more recent period, and a reduction in broad mixed digital health labeling. These findings support the interpretation that the final 2022–2025 corpus reflects a more specific and management-relevant subset of the broader 2018–2025 literature, while also acknowledging that earlier studies contribute important contextual evidence.

**Fig 4:**
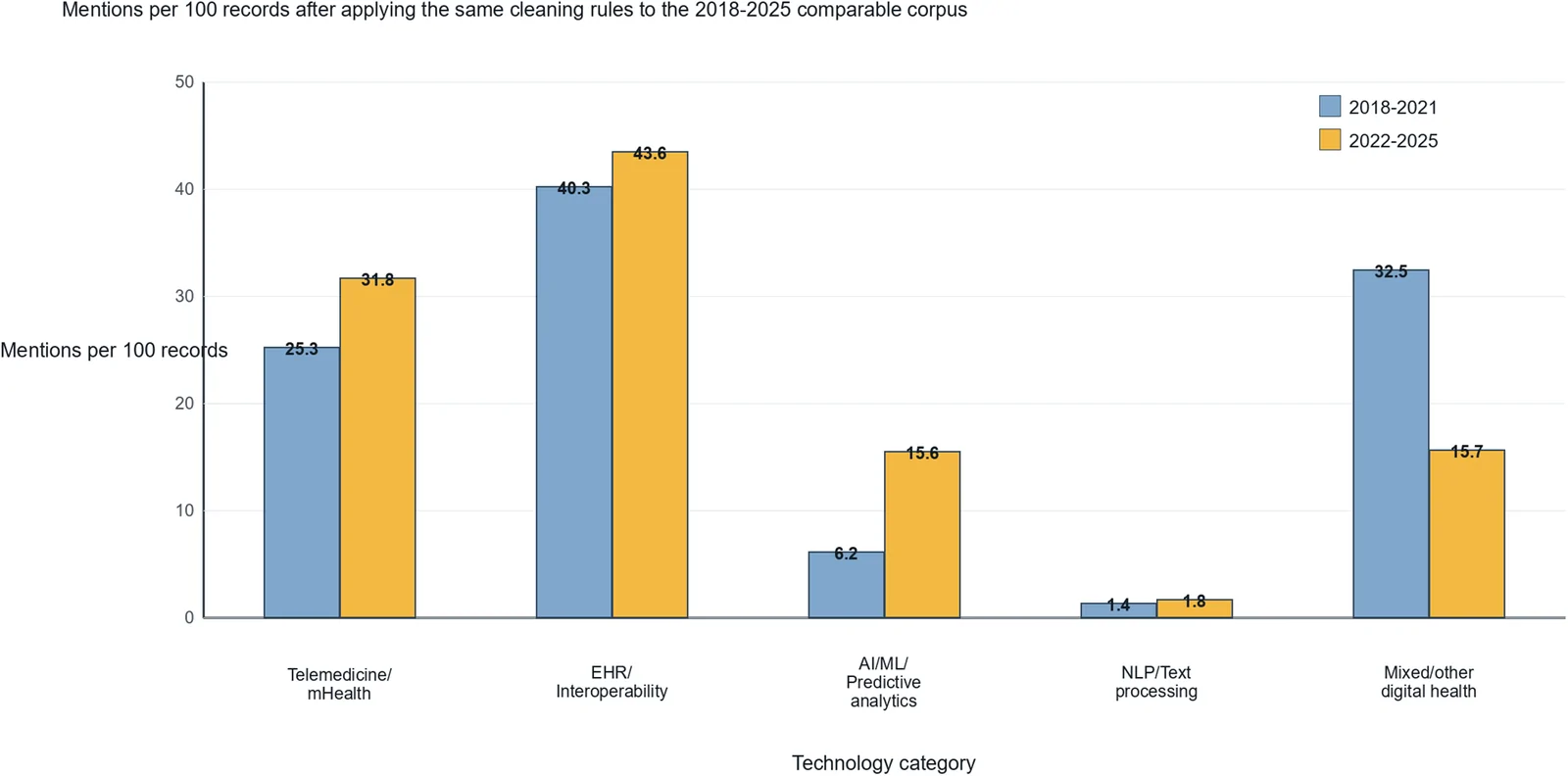
Temporal sensitivity analysis by technology category. Bars show technology-category mentions per 100 records in the cleaned comparable corpus for 2018–2021 and 2022–2025.

The expanded outcome-level temporal sensitivity analysis also showed that the post-2021 focus did not create a fundamentally different management-outcome profile. In the cleaned comparable corpus, operating-cost indicators decreased from 11.45 mentions per 100 records in 2018–2021 to 8.68 mentions per 100 records in 2022–2025, whereas patient/professional satisfaction remained nearly unchanged (6.84 vs. 6.87 mentions per 100 records). Waiting-time indicators increased modestly from 3.42 to 4.16 mentions per 100 records, and resource-utilization indicators increased from 2.22 to 3.98 mentions per 100 records. Administrative-efficiency indicators decreased from 4.44 to 3.25 mentions per 100 records. These results suggest continuity in the broad management-outcome structure, while confirming that the evidence remains too heterogeneous for temporal effect estimation. Table 8 summarizes the outcome-level sensitivity analysis.

**Table 8:** Temporal sensitivity analysis by management-outcome category.

Management-outcome category	2018–2021 mentions per 100 records	2022–2025 mentions per 100 records	Absolute difference
Operating costs	11.45	8.68	-2.77
Patient/professional satisfaction	6.84	6.87	+0.03
Administrative efficiency	4.44	3.25	-1.19
Waiting times	3.42	4.16	+0.74
Resource utilization	2.22	3.98	+1.76

### 3.5. Quality and risk-of-bias assessment

A stratified subsample of 40 studies from the final cleaned corpus was assessed using the JBI checklist domains to provide a validated risk-of-bias assessment alongside the descriptive synthesis [29]. The subsample was distributed across the main technology categories to avoid restricting the assessment to the dominant telemedicine/mHealth group. JBI domains were used because the final corpus included heterogeneous study designs, including observational, quasi-experimental, implementation, pilot, and mixed-methods studies. Although RoB 2 and ROBINS-I are widely used for randomized and non-randomized intervention studies, respectively [30,31], the JBI framework provides a flexible structure for appraising diverse empirical designs within a single review.

Because the assessment was conducted using abstract-level and metadata-level information, studies with incomplete reporting were flagged as requiring full-text confirmation rather than being interpreted as definitively high risk. The preliminary risk distribution showed that 19 studies (47.5%) were classified as moderate risk, 15 studies (37.5%) as low risk, and 6 studies (15.0%) as high or unclear risk. Thus, most studies in the stratified subsample were classified as low or moderate preliminary risk. The high/unclear category primarily reflected insufficient reporting of design, sampling, comparator, or outcome-measurement details in the available metadata, rather than confirmed evidence of methodological invalidity. Table 9 summarizes the preliminary JBI risk-of-bias distribution in the stratified subsample.

**Table 9:** Preliminary JBI risk-of-bias distribution in the stratified subsample (n = 40).

Preliminary risk classification	Studies (n)	Percentage
Moderate risk	19	47.5%
Low risk	15	37.5%
High/unclear risk	6	15.0%

The category-level distribution showed a moderate preliminary risk, with the largest group in telemedicine/mHealth studies: 10 moderate-risk, 3 low-risk, and 3 high/unclear-risk studies. EHR/interoperability studies were mostly classified as low or moderate preliminary risk, with 5 low-risk and 3 moderate-risk studies, and no high- or unclear-risk studies in the subsample. AI/ML and predictive analytics included 4 low-risk, 2 moderate-risk, and 2 high- or unclear-risk studies, indicating greater reporting variability in this category. Mixed or other digital health included 2 low-risk, 3 moderate-risk, and 1 high/unclear-risk study. NLP/text-processing studies were too few to support a stable category-level interpretation, with only 2 studies represented in the subsample Table 10 in S1 Text.

Fig 5 summarizes the preliminary JBI distribution in the stratified subsample. Panel A shows the overall distribution of low, moderate, and high/unclear risk classifications, while Panel B shows the distribution of these classifications across primary technology categories.

**Fig 5:**
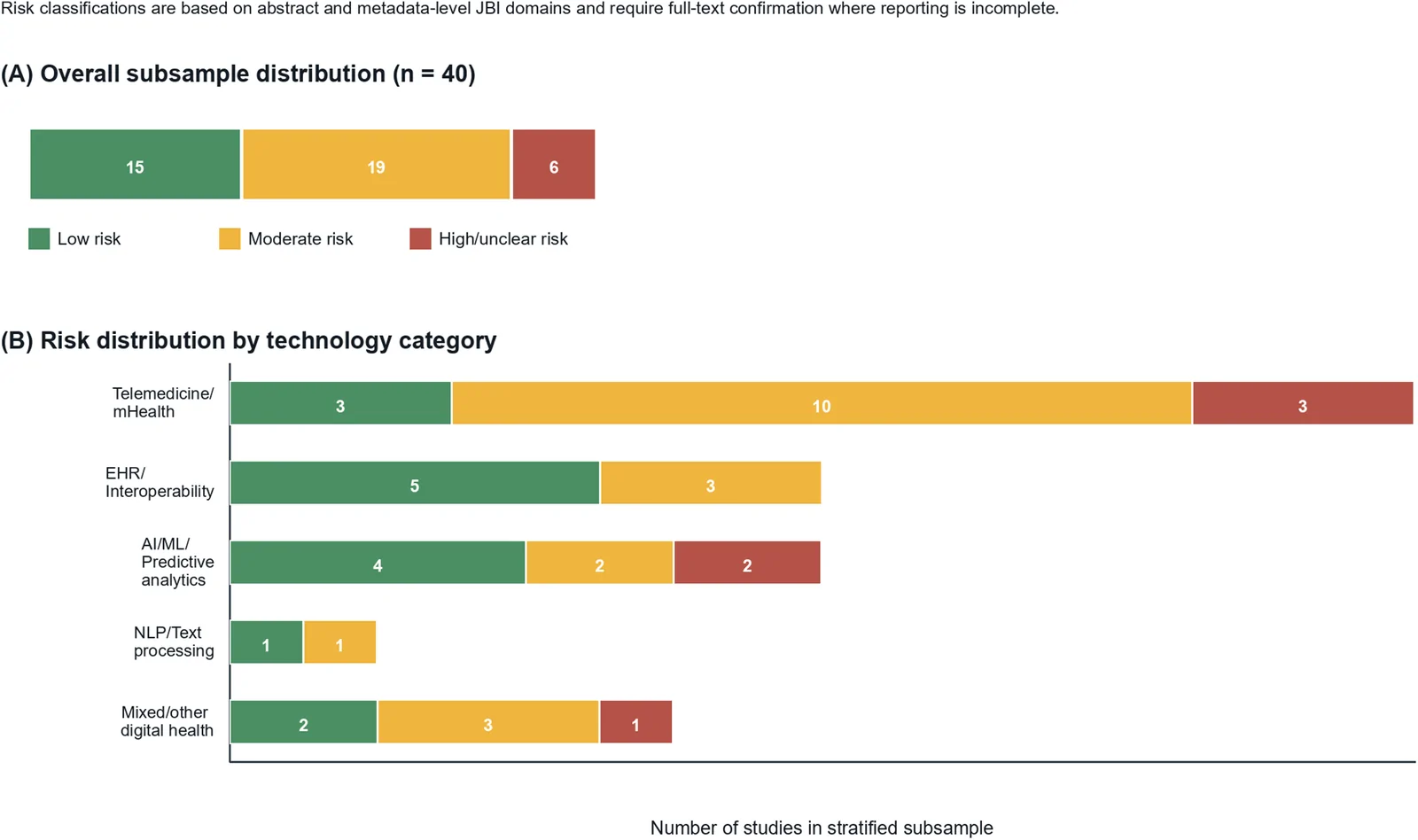
Preliminary JBI risk-of-bias check in the stratified subsample. Fig 5A shows the overall preliminary risk distribution. Fig 5B shows risk classification by primary technology category.

## 4. Discussion

This systematic review and descriptive quantitative synthesis examined the relationship between digital health technologies and management outcomes in primary care using a final cleaned corpus of 172 studies. The synthesis shows that the evidence base is concentrated mainly in telemedicine/mHealth, followed by EHR/interoperability and AI/ML or predictive analytics. Management indicators were reported unevenly across studies, with patient or professional satisfaction and operating costs appearing more frequently than waiting times, administrative efficiency, or resource utilization. This pattern suggests that the literature has advanced more rapidly in documenting service experience and economic dimensions than in producing standardized operational metrics suitable for inferential comparison Table 11 in S1 Data.

The decision to present a descriptive quantitative synthesis rather than a formal meta-analysis was supported by the structure of the evidence. Across the final corpus, studies differed substantially in design, intervention content, comparator structure, outcome definitions, and statistical reporting. Explicit reporting of statistical significance was uncommon for several management indicators, particularly waiting times and administrative efficiency. Therefore, the findings should be interpreted as evidence of technology-outcome alignment and reporting patterns, rather than as pooled estimates of intervention effect. This distinction is consistent with transparent reporting principles for systematic reviews, in which synthesis decisions should reflect the structure and comparability of the available evidence [22].

Telemedicine and mHealth formed the largest technology category in the final corpus. Their relationship with patient or professional satisfaction and operating costs was the most visible pattern in the technology-by-indicator matrix. This finding is consistent with studies showing that telehealth can support access, referral efficiency, continuity, staffing flexibility, and service reach when embedded in organized primary care workflows [1,2,23,55]. It is also consistent with evidence from rural, safety-net, and crisis-affected settings, where telemedicine has been used to extend care delivery and maintain access to services under constrained conditions [56–59]. However, the synthesis also shows that evidence on telemedicine remains unevenly reported from a management perspective. Satisfaction and cost outcomes were more frequently identified than administrative efficiency or waiting-time outcomes, suggesting that the operational impact of telemedicine is still often assessed using patient-facing or economic indicators rather than standardized workflow metrics.

The telemedicine evidence also illustrates the importance of implementation context. Studies of telemedicine integrated into traditional primary care, community health settings, free clinics, and health and wellness centers show that uptake and perceived usefulness depend on organizational readiness, digital access, user support, and local workflow adaptation [16,60,61]. Prior reviews and meta-analyses have synthesized telehealth effects in disease-specific or access-focused contexts, including escalation to emergency care, hypertension management, and cardiovascular risk management [19–21]. The present synthesis extends that perspective by focusing specifically on management indicators and by showing that the literature still reports operational outcomes unevenly across technology categories.

EHR/interoperability studies represented a smaller but structurally important component of the evidence base. Unlike telemedicine/mHealth, EHR/interoperability was distributed across multiple management indicators, including administrative efficiency, resource utilization, operating costs, and satisfaction. This pattern supports the interpretation that electronic records and interoperable systems function less as isolated interventions and more as enabling infrastructure for service coordination, data traceability, and workflow integration [4,9,33,62]. Studies on EHR-enabled pathways, patient portals, inbox workload, EHR implementation, and clinician communication tools show that digital infrastructure can reshape administrative work and clinical coordination [12,26,34,46]. From a management perspective, this suggests that the operational value of EHR systems depends not only on the digitization of records but also on their integration with patient portals, messaging systems, decision-support tools, and interprofessional coordination mechanisms.

Interoperability and data traceability also have implications for governance and institutional sustainability. Blockchain-enabled EHR systems, provenance-aware architectures, token-based security approaches, and oracle-based interoperability models illustrate how technical infrastructures are increasingly being designed around data integrity, traceability, and trusted exchange [24,63,64]. Although these approaches remain underrepresented in evaluations of primary care management, they point to a broader shift: digital health management depends on the reliability, interoperability, and governance of data, not solely on the availability of digital tools.

AI/ML and predictive analytics appeared as an emerging but less frequent domain. Their strongest links were with operating costs, administrative efficiency, and resource utilization, which is consistent with their expected role in demand forecasting, screening, triage support, decision support, and resource planning [11,13,35,48,52]. Nevertheless, the evidence remains methodologically limited. Many studies did not provide comparable validation metrics, external validation, prospective evaluation, or implementation-level assessment. Trust in AI-based triage and decision support remains an important implementation issue because model outputs may affect prioritization, professional judgment, and patient confidence [65]. These findings therefore support a cautious interpretation: AI/ML tools may contribute to management optimization, but their operational value depends on transparent model development, local validation, monitoring for drift, and evaluation of real-world workflow consequences.

The limited representation of NLP/text-processing studies is notable. Although NLP and ambient documentation tools are frequently discussed as mechanisms for structuring clinical information and reducing documentation burden, few studies in the final corpus linked these tools directly to measurable management outcomes in primary care. Existing work on ambient AI documentation and clinician perceptions of generative AI suggests potential relevance for documentation quality, workload, and administrative efficiency [36,37]. Future evaluations of NLP should move beyond technical performance metrics and assess whether text-processing tools reduce workload, improve documentation completeness, support care coordination, or generate reliable management indicators.

The temporal sensitivity analysis helps contextualize the final corpus’s post-2021 focus. The comparison between 2018–2021 and 2022–2025 did not show a reversal of the main technology profile. EHR/interoperability and telemedicine/mHealth remained prominent in both periods, while AI/ML increased in the more recent period, and mixed/other digital health labels decreased. This temporal pattern aligns with studies describing the expansion of telemedicine during and after the COVID-19 period, as well as more recent studies of integrated digital health applications and care models [49–51,53,54]. This suggests that the final 2022–2025 corpus reflects a more specific and operationally classifiable segment of the broader 2018–2025 literature rather than an entirely different evidence base.

The findings also highlight the importance of data governance across health systems. Digital health maturity cannot be interpreted independently of privacy regulation, interoperability policy, data-sharing rules, and institutional capacity. The international scope of the evidence base implies substantial variation in legal and operational conditions governing the implementation of EHRs, telemedicine platforms, AI tools, and interoperable data exchange. In resource-limited or infrastructure-constrained settings, digital transformation may be limited by connectivity, workforce training, procurement capacity, data standards, and continuity of technical support. Evidence from community health worker-led interventions, digital health initiatives in Bangladesh, and Southeast Asian digital health reviews illustrates how implementation capacity and local infrastructure shape digital health performance [17,27,66]. These contextual factors should be considered when interpreting management outcomes, because a technology that improves efficiency in a highly integrated health system may not produce the same effect in settings with fragmented infrastructure or limited data governance capacity.

This governance issue is particularly relevant for privacy-preserving AI and cross-institutional data reuse. Federated learning has been proposed as a way to train models across distributed health data environments without moving patient-level data into a single repository, but it also introduces challenges related to technical standardization, governance accountability, model monitoring, and validation across heterogeneous sites [67]. For primary care management, this means that data-sharing strategies must be evaluated not only by their predictive performance but also by their ability to preserve privacy, support reproducibility, and remain operationally feasible across differently resourced health systems.

Algorithmic bias and data imbalance are especially relevant for AI/ML and NLP applications in primary care management. Predictive analytics and text-processing systems may reproduce inequities when training data underrepresent rural populations, minority groups, low-resource settings, or patients with incomplete digital records. Bias may also arise from differential access to telemedicine, uneven documentation quality, language variation, or missing data in EHR systems. Studies on digital divide interventions, language preference, telemedicine access, and digital exclusion show that digital health implementation can interact with social and structural inequities [5,8,14,15,68]. The current literature has rarely measured these risks as management outcomes. Future evaluations should therefore include bias-aware metrics, subgroup performance reporting, calibration across demographic and service-context groups, and monitoring of whether algorithmic tools alter access, workload, or resource allocation unequally across populations.

Evidence from population-management algorithms shows that bias can arise not only from model architecture but also from the choice of proxy outcomes, such as using health care cost as a surrogate for clinical need when access to care is unequal across groups [69]. This is directly relevant to management-oriented digital health research because operational indicators such as utilization, cost, scheduling priority, and workload may encode structural inequities if they are treated as neutral ground truth. Bias-aware evaluation should therefore examine proxy selection, subgroup calibration, missingness patterns, and whether algorithmic recommendations redistribute access or administrative burden unequally.

Medical data should be understood as a strategic institutional asset rather than only as a by-product of care delivery. The value of data for management depends on quality, interoperability, governance, ethical use, and the capacity to transform data into actionable operational intelligence. Recent conceptual work on medical data assetization emphasizes the need for trusted data spaces, lifecycle governance, stakeholder accountability, and ethical safeguards when using health data for decision-making and innovation [18]. This perspective is particularly relevant for predictive analytics in primary care, where local sample sizes may be small, outcome events may be sparse, and model performance may be unstable across subgroups. In such settings, data-sharing frameworks, federated approaches, standardized metadata, and external validation are essential to avoid overfitting and to improve generalizability.

For AI-based decision-support systems, reporting and evaluation frameworks such as DECIDE-AI emphasize that early-stage clinical evaluation should consider not only model accuracy but also human factors, workflow integration, safety, failure modes, and implementation context [70]. These requirements align with the main finding of this review: management outcomes cannot be inferred from technical performance alone. Future primary care studies should therefore report how AI tools are introduced into actual workflows, how users interact with them, and whether they change administrative efficiency, resource allocation, access, or workload in measurable ways.

The risk-of-bias subsample assessment provides additional context for interpreting the evidence. Most studies in the stratified JBI subsample were classified as low or moderate preliminary risk. Still, a smaller group remained high or unclear due to incomplete reporting of design, sampling, comparator, measurement, or analysis details. This finding reinforces the need for cautious interpretation. The issue is not simply whether digital health interventions appear beneficial, but whether the available studies report methods and outcomes with enough consistency to support transferable management conclusions. Hybrid effectiveness-implementation protocols, pragmatic trials, longitudinal implementation studies, and real-world evaluations provide useful directions for strengthening this evidence base [71–80].

Implementation frameworks also help explain why digital health technologies may fail to produce consistent management outcomes across settings. The NASSS framework highlights that nonadoption, abandonment, scale-up, spread, and sustainability depend on the interaction between the condition, technology, value proposition, adopter system, organization, wider context, and embedding over time [81]. This perspective supports interpreting the heterogeneity observed in this review as an implementation and system-capacity issue, rather than only as a statistical or reporting problem.

Several limitations should be considered. First, although targeted full-text verification was performed for the records contributing directly to the management-outcome synthesis, the broader 172-record corpus still relied on structured bibliographic, abstract, and metadata fields for general corpus characterization. The full-text verification was therefore designed to strengthen outcome-level claims, not to convert the entire review into a complete full-text extraction study across all 172 records. Second, the heterogeneity in designs, interventions, and indicators precluded a formal meta-analysis and limited the ability to estimate pooled effects. Third, the JBI assessment was conducted in a stratified subsample rather than across all included studies and should therefore be interpreted as a validated risk-of-bias check rather than a complete article-level appraisal of the entire corpus. Fourth, the review included English-language peer-reviewed literature, which may underrepresent regional or non-English implementation experiences. Finally, although the temporal sensitivity analysis reduced concerns about the post-2021 analytical focus, it does not rule out the possibility that earlier studies provide contextual insights not captured in the final synthesis.

Future research should prioritize standardized management indicators for digital primary care, including waiting times, administrative workload, resource utilization, operating costs, continuity of care, and patient and professional experience. Studies should report comparable denominators, follow-up periods, effect estimates, measures of uncertainty, and subgroup analyses. For AI/ML and NLP, future work should include external validation, explainability, bias assessment, and prospective monitoring in real-world workflows. For telemedicine and EHR/interoperability, research should move beyond adoption and satisfaction metrics toward integrated evaluations of workload, equity, cost, safety, and sustainability across diverse health-system contexts.

## 5. Conclusions and future work

This systematic review provides a descriptive quantitative synthesis of the evidence linking digital health technologies to management outcomes in primary care. Based on a cleaned corpus of 172 studies, the findings show that the current evidence base is concentrated mainly in telemedicine/mHealth, EHR/interoperability, and AI/ML or predictive analytics. The most frequently reported management indicators were patient or professional satisfaction and operating costs, whereas waiting times, administrative efficiency, and resource utilization were reported less consistently. These findings suggest that digital health research in primary care has advanced in documenting service experience and economic dimensions, but remains limited by heterogeneous outcome definitions and uneven statistical reporting.

Telemedicine and mHealth represented the largest technology category in the final corpus. The descriptive synthesis showed that these interventions were most often linked to satisfaction and operating-cost indicators, reflecting their role in expanding access, supporting remote follow-up, and reorganizing care delivery. However, the evidence does not support uniform causal claims across settings. Future studies should evaluate telemedicine and mHealth using standardized operational metrics, including waiting times, professional workload, continuity of care, cost per encounter, and equity of access.

EHR/interoperability emerged as a structurally important domain for digital primary care management. Although less frequent than telemedicine/mHealth, this category was distributed across multiple management indicators, suggesting that electronic records and interoperable systems may function as enabling infrastructure for coordination, traceability, and workflow integration. Future work should examine not only whether EHR systems are implemented, but also how interoperability, terminology standards, patient portals, and interprofessional communication tools affect administrative burden, care coordination, and resource use.

AI/ML and predictive analytics emerged as an area with potential relevance to scheduling, demand forecasting, triage support, and resource planning. Nevertheless, the current evidence remains limited by inconsistent validation, incomplete reporting, and a scarcity of prospective implementation studies. Future research should prioritize external validation, bias assessment, explainability, calibration across population subgroups, and monitoring of workflow consequences after implementation. NLP/text-processing applications were rare in the final corpus, indicating an important gap in evidence linking documentation automation, inbox management, coding support, or information extraction to measurable primary care management outcomes.

The synthesis also highlights a methodological gap in the field. Many studies did not report comparable effect estimates, uncertainty measures, or standardized management indicators, which prevented formal meta-analysis. The expanded temporal sensitivity analysis suggested that the post-2021 corpus did not fundamentally reverse either the broader technology profile or the distribution of management-outcome categories, although the more recent period showed greater specificity in technology classification and stronger representation of AI/ML. The JBI subsample risk-of-bias check indicated that most assessed studies had a low or moderate preliminary risk. In contrast, a smaller group had a high or unclear risk due to incomplete reporting. These findings reinforce the need for stronger study designs, clearer reporting, and validated evaluation frameworks.

Future work should move toward harmonized outcome sets for digital primary care management. Core indicators should include waiting times, administrative efficiency, resource utilization, operating costs, continuity of care, patient and professional satisfaction, and equity-related outcomes. Studies should report denominators, follow-up periods, effect estimates, confidence intervals, and subgroup analyses wherever possible. Economic evaluations, implementation studies, and multicenter pragmatic designs will be particularly important for determining whether digital health interventions produce transferable management benefits across different health-system contexts.

Digital health in primary care should be evaluated not only as a set of isolated tools but as an integrated management infrastructure shaped by data governance, interoperability, workforce capacity, and equity safeguards. The next phase of research should therefore combine health services evaluation, implementation science, informatics, and responsible AI assessment to determine how digital technologies can support efficient, equitable, and sustainable primary care systems.

## Supporting information

S1 PRISMA Checklist PRISMA 2020 checklist. The completed PRISMA 2020 checklist is provided as a supporting information file. (DOCX)

S1 Text Detailed search strategies. The complete search strategies used for each database are provided below. These include Boolean operators, controlled vocabulary where available (MeSH and CINAHL Headings), and free-text terms adapted to the syntax of each source. Filters for publication period, English language, document type, and abstract availability were applied according to the options available in each database. The final documented export and corpus consolidation date was October 7, 2025.
(RAR)

S1 Data Full-text verification and management-outcome extraction matrix. The accompanying workbook S1_Data.xlsx provides the record-level verification matrix for the 53 studies that contributed directly to the management-outcome synthesis. The workbook includes the study identifier, bibliographic fields, management-outcome coding, full-text/PDF matching status, full-text extraction diagnostics, indicator-support flags, statistical-language detection, and a codebook describing the operational definitions used during verification. Table 11 summarizes the scope and outputs of this verification process.
(XLSX)
