# Supplementary material for: Digital health technologies and measurable management outcomes in primary care: A systematic review, descriptive quantitative synthesis, and temporal sensitivity analysis
Source: PLOS Digit Health. 2026 Nov 4;5(11):e0001778. doi: 10.1371/journal.pdig.0001778 (PMC13734591; doi:10.1371/journal.pdig.0001778)
Supplement: Supplementary file 1 [file pdig.0001778.s001.docx]

| **Section and Topic** | **Item #** | **Checklist item** | **Location where item is reported** |
| --- | --- | --- | --- |
| **TITLE** | | |  |
| Title | 1 | Identify the report as a systematic review. | Title page 1 |
| **ABSTRACT** | | |  |
| Abstract | 2 | See the PRISMA 2020 for Abstracts checklist. | Abstract, page 1 |
| **INTRODUCTION** | | |  |
| Rationale | 3 | Describe the rationale for the review in the context of existing knowledge. | Introduction, section 1, page 2 |
| Objectives | 4 | Provide an explicit statement of the objective(s) or question(s) the review addresses. | Introduction, last paragraph, page 2–3 |
| **METHODS** | | |  |
| Eligibility criteria | 5 | Specify the inclusion and exclusion criteria for the review and how studies were grouped for the syntheses. | Methods, subsection, Review Design (criteria definition and grouping logic). |
| Information sources | 6 | Specify all databases, registers, websites, organisations, reference lists and other sources searched or consulted to identify studies. Specify the date when each source was last searched or consulted. | Information Sources & Search Strategy (PubMed, Scopus, Web of Science, CINAHL, IEEE Xplore). |
| Search strategy | 7 | Present the full search strategies for all databases, registers and websites, including any filters and limits used. | Information Sources & Search Strategy; Listing 1: PubMed search string used in systematic review. |
| Selection process | 8 | Specify the methods used to decide whether a study met the inclusion criteria of the review, including how many reviewers screened each record and each report retrieved, whether they worked independently, and if applicable, details of automation tools used in the process. | Study selection process (automated screening, duplicate removal, manual verification). |
| Data collection process | 9 | Specify the methods used to collect data from reports, including how many reviewers collected data from each report, whether they worked independently, any processes for obtaining or confirming data from study investigators, and if applicable, details of automation tools used in the process. | Data extraction (data collected by two reviewers independently using an automated extraction script developed in Python, followed by manual validation for consistency and completeness). |
| Data items | 10a | List and define all outcomes for which data were sought. Specify whether all results that were compatible with each outcome domain in each study were sought (e.g. for all measures, time points, analyses), and if not, the methods used to decide which results to collect. | Data extraction (management indicators: cost efficiency, administrative performance, resource utilization, user satisfaction). |
|  | 10b | List and define all other variables for which data were sought (e.g. participant and intervention characteristics, funding sources). Describe any assumptions made about any missing or unclear information. | Data extraction (context, technology type, intervention model, publication metadata). |
| Study risk of bias assessment | 11 | Specify the methods used to assess risk of bias in the included studies, including details of the tool(s) used, how many reviewers assessed each study and whether they worked independently, and if applicable, details of automation tools used in the process. | Quality and Risk of Bias Analysis (bias typology and quality scoring). |
| Effect measures | 12 | Specify for each outcome the effect measure(s) (e.g. risk ratio, mean difference) used in the synthesis or presentation of results. | Review Design and Quantitative analysis (aggregated proportions and statistical synthesis). |
| Synthesis methods | 13a | Describe the processes used to decide which studies were eligible for each synthesis (e.g. tabulating the study intervention characteristics and comparing against the planned groups for each synthesis (item #5)). | Study selection process and Review Design (studies classified into four synthesis groups based on digital health technology type: telemedicine/mHealth, EHR/interoperability, AI/predictive analytics, and NLP/word processing; criteria aligned with inclusion variables defined in Review Design). |
|  | 13b | Describe any methods required to prepare the data for presentation or synthesis, such as handling of missing summary statistics, or data conversions. | Data extraction (automated text mining used to standardize terminology, convert frequency data into proportional indicators, and normalize publication metadata; missing variables cross-checked manually). |
|  | 13c | Describe any methods used to tabulate or visually display results of individual studies and syntheses. | Results (visual synthesis through thematic clustering, bar and network plots, and proportional matrices; figures generated via Python visualization scripts referenced in the Zenodo dataset). |
|  | 13d | Describe any methods used to synthesize results and provide a rationale for the choice(s). If meta-analysis was performed, describe the model(s), method(s) to identify the presence and extent of statistical heterogeneity, and software package(s) used. | Review Design and Quantitative evidence synthesis (synthesis based on weighted aggregation of frequency distributions and quantitative descriptors; no traditional meta-analysis performed due to heterogeneity of outcomes; rationale: descriptive quantitative synthesis to preserve comparability across management indicators). |
|  | 13e | Describe any methods used to explore possible causes of heterogeneity among study results (e.g. subgroup analysis, meta-regression). | Quantitative evidence synthesis (heterogeneity explored by grouping studies according to technology domain, regional context, and methodological rigor, analyzed through comparative proportions and distributional contrasts). |
|  | 13f | Describe any sensitivity analyses conducted to assess robustness of the synthesized results. | Quantitative evidence synthesis (sensitivity analysis through exclusion of low-quality or incomplete studies and recalculation of aggregated indicators to verify stability of outcomes). |
| Reporting bias assessment | 14 | Describe any methods used to assess risk of bias due to missing results in a synthesis (arising from reporting biases). | Quality and Risk of Bias Analysis (reporting bias assessed through consistency checks between described outcomes and reported metrics; completeness validated by cross-referencing extracted variables with study abstracts and methods). |
| Certainty assessment | 15 | Describe any methods used to assess certainty (or confidence) in the body of evidence for an outcome. | Quality and Risk of Bias Analysis (certainty evaluated through the proportion of studies rated as high methodological quality and consistent outcome reporting; global confidence level summarized in the distribution chart of Figure 5). |
| **RESULTS** | | |  |
| Study selection | 16a | Describe the results of the search and selection process, from the number of records identified in the search to the number of studies included in the review, ideally using a flow diagram. | Results: Study selection and Figure 1 (PRISMA 2020 Flow Diagram) total of 256 included studies after screening of 4,870 records and exclusion of duplicates, non-primary studies, and non-quantitative reports. |
|  | 16b | Cite studies that might appear to meet the inclusion criteria, but which were excluded, and explain why they were excluded. | Results → Identification and selection of studies (initial paragraphs, table “Databases consulted, coverage, and retrieved records”) and Figure 1, PRISMA 2020 flowchart. |
| Study characteristics | 17 | Cite each included study and present its characteristics. | Results: Descriptive and Quantitative Results. Summary tables include the year, technology domain, region, and study design; individual citations appear in the reference list, linked to each synthesis cluster. |
| Risk of bias in studies | 18 | Present assessments of risk of bias for each included study. | Quality and Risk of Bias Analysis: distributions of methodological quality are presented as categorical proportions (low, moderate, and high risk). Supporting details are provided in Table 12 (Overall Assessment of Quality and Risk of Bias), which shows the proportion of bias categories per technology domain. |
| Results of individual studies | 19 | For all outcomes, present, for each study: (a) summary statistics for each group (where appropriate) and (b) an effect estimate and its precision (e.g. confidence/credible interval), ideally using structured tables or plots. | Results, the Quality and Risk of Bias Analysis section presents aggregated findings by management indicator (cost efficiency, administrative performance, resource utilization, and satisfaction). Frequency and proportions are visualized by technology domain. Confidence intervals were not computed due to metric heterogeneity across included studies |
| Results of syntheses | 20a | For each synthesis, briefly summarise the characteristics and risk of bias among contributing studies. | Results → Synthesis of findings (table “Thematic distribution of studies across identified clusters (n = 256)”, Figure 3) and cross-reference with Quality and Risk of Bias Analysis (distributions by technology in the risk table). |
|  | 20b | Present results of all statistical syntheses conducted. If meta-analysis was done, present for each the summary estimate and its precision (e.g. confidence/credible interval) and measures of statistical heterogeneity. If comparing groups, describe the direction of the effect. | Results → Impact on management indicators (numerical summary aggregated by indicator and by technology; see Figure 4). Meta-analysis: not performed due to the heterogeneity of the outcomes/measures. The direction of the effect is described (e.g., telemedicine/mHealth → costs↓, satisfaction↑; EHR → administrative efficiency↑). |
|  | 20c | Present results of all investigations of possible causes of heterogeneity among study results. | Results → Synthesis of findings and Impact on management indicators (variation by technological domain, design and period; Figures 2 and 4 show differences in coverage versus significant evidence). |
|  | 20d | Present results of all sensitivity analyses conducted to assess the robustness of the synthesized results. | No sensitivity analysis reported (no formal sensitivity analysis is reported in the manuscript). |
| Reporting biases | 21 | Present assessments of risk of bias due to missing results (arising from reporting biases) for each synthesis assessed. | Results → Quality and Risk of Bias Analysis (dominant biases such as measurement and confounding are identified; missing data indicated; reporting bias not formally assessed; acknowledged in the discussion of limitations). |
| Certainty of evidence | 22 | Present assessments of certainty (or confidence) in the body of evidence for each outcome assessed. | Results → Quality and Risk of Bias Analysis (overall profile with a predominance of moderate risk; Figure 5); expanded in Discussion (intermediate technical maturity and methodological heterogeneity). GRADE or another formal scheme: not applied. |
| **DISCUSSION** | | |  |
| Discussion | 23a | Provide a general interpretation of the results in the context of other evidence. | Discussion, opening three paragraphs. Interpret the results of the 256 studies and compare them with previous evidence on telemedicine, EHR, and AI. Cite relevant previous work and contextualize the contribution with respect to previous reviews focused on clinical effectiveness. |
|  | 23b | Discuss any limitations of the evidence included in the review. | Discussion, final two paragraphs before “The review presents limitations…”. Identifies limitations of the primary studies, including methodological heterogeneity, lack of cross-validation, and the predominance of observational designs; also addresses publication bias and contextual variability. |
|  | 23c | Discuss any limitations of the review processes used. | Discussion, final paragraph (beginning “The review presents limitations inherent to the heterogeneity of the corpus analyzed”). It acknowledges limitations of the review process itself, including diversity in design, language, and methodological quality, which restricts inferential capacity. |
|  | 23d | Discuss implications of the results for practice, policy, and future research. | Discussion, middle, and concluding paragraphs. It details the practical and public policy implications (EHR-telemedicine integration, interoperability, digital literacy, equity) and future directions (standardization of metrics, multicenter trials, validation of explainable AI, blockchain in traceability). |
| **OTHER INFORMATION** | | |  |
| Registration and protocol | 24a | Provide registration information for the review, including register name and registration number, or state that the review was not registered. | Methods, Review Design paragraph. The review was registered in PROSPERO (ID: CRD420241174575) on 22 October 2025, titled “Linking Digital Health Maturity to Measurable Management Outcomes in Primary Care: A Systematic Review and Quantitative Evidence Synthesis.” The record is awaiting co-author confirmation for public release. |
|  | 24b | Indicate where the review protocol can be accessed, or state that a protocol was not prepared. | The protocol will be accessible at https://www.crd.york.ac.uk/prospero/display_record.php?ID=CRD420241174575 |
|  | 24c | Describe and explain any amendments to information provided at registration or in the protocol. | No amendments have been made since submission; the final methodology remains unchanged from the registered version. |
| Support | 25 | Describe sources of financial or non-financial support for the review, and the role of the funders or sponsors in the review. | Acknowledgments / Funding statement (PLOS submission field). The authors declare no financial support; the review was conducted as independent academic research under the Universidad de Las Américas (Ecuador). |
| Competing interests | 26 | Declare any competing interests of review authors. | Competing Interests Statement. The authors declare no competing interests. |
| Availability of data, code and other materials | 27 | Report which of the following are publicly available and where they can be found: template data collection forms; data extracted from included studies; data used for all analyses; analytic code; any other materials used in the review. | Data Availability Statement. All data extraction templates, datasets, and analytic scripts are openly available in Zenodo (DOI: 10.5281/zenodo.17544090). No proprietary or restricted data were used. |

*From:*  Page MJ, McKenzie JE, Bossuyt PM, Boutron I, Hoffmann TC, Mulrow CD, et al. The PRISMA 2020 statement: an updated guideline for reporting systematic reviews. BMJ 2021;372:n71. doi: 10.1136/bmj.n71. This work is licensed under CC BY 4.0. To view a copy of this license, visit <https://creativecommons.org/licenses/by/4.0/>
